# TNFR1 is associated with short-term mortality in patients with diabetes and acute dyspnea seeking care at the emergency department

**DOI:** 10.1007/s00592-020-01527-3

**Published:** 2020-04-12

**Authors:** P. Wändell, A. C. Carlsson, A. Larsson, O. Melander, T. Wessman, J. Ärnlöv, T. Ruge

**Affiliations:** 1 Department of Neurobiology, Care Sciences and Society, Karolinska Institutet, Huddinge, Sweden; 2 Academic Primary Health Care Centre, Stockholm Region, Stockholm, Sweden; 3 Department of Medical Sciences, Uppsala University, Uppsala, Sweden; 4 Department of Emergency and Internal Medicine, Skånes University Hospital, Malmö, Sweden; 5 Department of Clinical Sciences Malmö, Lund University, Lund, Sweden; 6 Department of Internal Medicine, Skåne University Hospital, Malmö, Sweden; 7 School of Health and Social Studies, Dalarna University, Falun, Sweden

**Keywords:** Diabetes, Soluble tumor necrosis factor alpha (TNFR), Mortality, Triage level

## Abstract

**Background:**

Circulating levels of TNF alpha receptor 1 (TNFR1) and 2 (TNFR2) are associated with increased long-term mortality and impaired kidney function.

**Aim:**

To study association between circulating levels of TNFR1 and TNFR2 and short-term mortality in patients with diabetes and dyspnea.

**Population and methods:**

Patients aged ≥ 18 years seeking at emergency department (ED) during daytime on weekdays between December 2013 and July 2018, with diabetes and acute dyspnea, identified at the triage process, were included. Participants (*n* = 291) were triaged according to Medical Emergency Triage and Treatment System-Adult score, and blood samples were collected. Association between TNFR1 and TNFR2, respectively, and 90-day mortality were estimated by Cox regression models adjusted for age, sex, BMI, creatinine and CRP.

**Results:**

Univariate models showed significant associations between TNFR1 and TNFR2, respectively, and CRP, age and creatinine. TNFR1 and TNFR2 tended to be elevated in patients with the highest triage level, compared to patients with lower triage levels (ns). In longitudinal analyses, TNFR1 but not TNFR2 was associated with increased short-term mortality, HR adjusted for age, BMI and creatinine 1.43 (95% CI 1.07–1.91), but not in the model also adjusted for CRP, HR 1.29 (95% CI 0.94–1.77). In secondary analysis for quartile 4 versus quartiles 1–3 of TNFR1, corresponding HRs were 2.46 (95% CI 1.27–5.15) and 2.21 (95% CI 1.07–2.56).

**Conclusions:**

We found a trend for the association between circulating TNFR1 levels and short-term mortality in patients with diabetes and acute dyspnea at the ED, possibly suggesting an inflammatory pathway for the association**.**

**Electronic supplementary material:**

The online version of this article (10.1007/s00592-020-01527-3) contains supplementary material, which is available to authorized users.

## Introduction

Tumor necrosis factor alpha (TNF alpha) is produced by macrophages/monocytes during acute inflammation and is involved in many signaling events within cells through tumor necrosis receptors (TNFR), mainly leading to necrosis or apoptosis [1]. TNFR1 is expressed on most cells, and the signaling pathway by TNF via TNFR1 mainly triggers pro-inflammatory pathways [2]. Membrane TNF and TNFR are expressed on the surface of cells, i.e., as a transmembrane protein, and cleaving of TNFR leads to liberated and soluble TNFR of two types: TNFR1 and TNFR2 [3]. Higher circulating TNFR1 independently predicts the progression to a worse GFR category and CKD incidence in elderly individuals [4] and also to a higher all-cause mortality risk, including both cancer and cardiovascular mortality, among individuals with both high and low levels of systemic inflammation [5], especially among elderly. Particularly, in patients with diabetes, TNFR1 and TNFR2 predict both a worsened kidney function and mortality [6, 7].

Earlier studies evaluating the TNFRs in patients with diabetes were based on epidemiological cohorts from the general population as well as to defined minor cohorts of patients with diabetes. To our knowledge, the association between TNFR and acute ill patients with diabetes is sparsely studied. Acute illness is often associated with higher levels of inflammation, and our hypothesis was that TNFR had the ability to predict also short-term mortality in this setting. We therefore investigated the association between levels of circulating TNFR1 and TNRF2 and short-term mortality in patients with diabetes and dyspnea admitted to the emergency department. Our hypothesis was that TNFR1 and TNRF2 would be associated with an increased mortality risk in these patients.

## Methods

### Study population

The study was conducted at the ED of Skåne University Hospital in Malmö (SUS Malmö), which is responsible for a catchment area of nearly 400,000 people with up to 85,000 visits per year. Patients 18 years of age and older who presented to the ED during daytime on weekdays, 8:00 a.m. to 5:00 p.m., with acute dyspnea between March 6, 2013, and July 1, 2018, were approached by a research nurse and were offered to take part in the study. Participants were triaged according to Medical Emergency Triage and Treatment System-Adult score (METTS-A) where after blood samples were collected [8]. A total of 291 patients with diabetes (type 1 or 2) were included.

### Clinical parameters

Blood pressure, oxygen saturation and heart rate were recorded by an automated oscillometer (CARESCAPE Monitor B850 or B650, General Electric Healthcare); consciousness was determined according to the Reaction Level Scale [9].

After inclusion, patients were questioned regarding smoking habits and categorized as non-smokers, former smokers (cessation one month ago or longer) or active smokers (regularly smoking the past month or longer), disease history associated with dyspnea (i.e., congestive heart failure, chronic obstructive pulmonary disease, asthma, coronary artery disease, atrial fibrillation, restrictive lung disease, cancer, thromboembolic disease or rheumatic disease) and current medications. The research nurses reviewed the patient journals in order to confirm the details, with the support of senior physicians whenever uncertainties occurred. Originally, METTS-A uses a five clinical priority levels with increasing clinical priority: blue (the lowest clinical priority—not life threatening), green, yellow, orange and red (the highest clinical priority—life threatening). The lowest clinical priority level blue was not used in the clinical triage of the patients included here due to the local triage routines; the lowest clinical triage priority here was green.

### Blood analyses

High-sensitivity plasma CRP was analyzed by a particle-enhanced turbidimetric assay, and plasma creatinine and lactate were analyzed by an IDMS-calibrated enzymatic assay [10]. The methods are accredited by Swedac, are included in the Swedish external quality assurance program for CRP and creatinine, respectively, and are routine methods. Within an hour of presentation at ED, blood was sampled, serum and plasma separated and subsequently put away for storage at − 80 °C for future analysis. TNFR1 and TNFR2 were analyzed by commercial sandwich ELISA kits (DY225 and DY726, R&D Systems, Minneapolis, MN, USA). The samples were analyzed as singletons in the study. Recombinant TNFR1 and TNFR2 were used as calibrators. The assays had a total coefficient of variation of approximately 6%. The assays were performed blinded without knowledge of clinical data.

### Statistical analysis

Baseline values are given as number or percentages, mean values with standard deviation (SD) or median values (for TNFR1 and TNFR2). Univariate linear regression analyses were used to study the association between TNFRs, with patient clinical characteristics at baseline such as age, BMI, blood pressure, respiratory rate, CRP, lactate, glucose and creatinine. Logarithmic TNFR1, TNFR2 and CRP were used in the regression analyses. TNFR1 and TNFR2 median values for the METTS-A groups were calculated by ANOVA. The association between TNFR1 and TNFR2 and 90-day mortality were analyzed using Cox proportional hazard models, for one SD increase in TNFR1 and TNFR2, and in secondary analyses also for the highest quartile of TNFR1 vs the quartiles 1–3. We used four models, i.e., Model A adjusted for age and sex, Model B as Model A but adjusted for BMI and creatinine and Model C as Model B but also adjusted for CRP. Missing data points (No), TNFR1 = 2, TNFR2 = 3, creatinine = 4, BMI = 10 and CRP = 5 were imputed. Results are expressed as pooled data. The follow-up time stretched from time of presentation at the ED to death within or end of follow-up. A *p* value of < 0.05 was considered statistically significant. Statistical analyses were performed with IBM SPSS statistics 25 (SPSS Inc., Chicago, IL).

## Results

### Study population

The baseline characteristics of the participants are presented in Table 1. Patients showed mean age of 74 years, with 56% men.

**Table 1:** Baseline characteristics for patients with diabetes seeking emergency department care for dyspnea

Baseline characteristic	
Female	44%
Age at survey (years)	74 (14)
Body mass index (kg/m^2^)	30 (7)
Systolic blood pressure (mmHg)	148 (28)
Diastolic blood pressure (mmHg)	81 (17)
Respiratory rate (frequency)	26 (7)
C-reactive protein (CRP, mg/L)	13.0 (31.2)
Lactate (mmol/L)	2.1 (1.1)
Glucose level (mmol/L)	11 (6)
Creatinine (µmol/L)	126 (111)
Circulating TNF alpha receptor 1 (pg/mL)	2804 (2678)
Circulating TNF alpha receptor 2 (pg/mL)	7589 (5740)
*METTS-A triage*	
Red—most acute (%)	16
Orange (%)	32
Yellow (%)	49
Green—least acute (%)	4
Admitted to hospital care (%)	69
90-day mortality (%)	14

Missing data points were less than 4% for all included characteristics except for diastolic blood pressure and lactate which were around 8% of data points. Mean values (with SD), but median values (with IQR) for TNF alpha receptor values and CRP

### Cross-sectional analyses between TNFRs and clinical characteristics

Table 2 shows linear regression analyses for TNFR1 and TNFR2, respectively, with the highest r^2^ for creatinine and CRP. ANOVA showed significantly higher levels of TNFR1 and TNFR2 in the red METTS-A group (highest priority), compared to lower triage priority groups. (Table 3). The 90-day mortality showed a U-formed association regarding mortality in relation to triage group (Table 3), with 27.7% for red priority, 11.8% for orange, 9.9% for yellow and 20.0% for green.

**Table 2:** Association between TNFR1, TNFR2 and baseline characteristics. Logarithmic TNFR1, TNFR2 and CRP were used only for regression analyses

Variable	TNFR1 (B (95% CI), r^2^)	TNFR2 (B, 95% CI, r^2^)
Sex	0.048 (− 0.12; 0.21), 0.001	0.07 (− 0.68; 0.21), 0.004
Age at survey (years)	0.009 (0.003; 0.015), 0.03**	0.005 (0.000; 0.01), 0.01
Body mass index (kg/m^2^)	0.003 (− 0.009; 0.015), 0.001	0.003 (− 0.008; 0.014), 0.001
Systolic blood pressure (mmHg)	− 0.003 (− 0.005; − 0.000), 0.01	− 0.002 (− 0.005; 0.000), 0.001
Diastolic blood pressure (mmHg)	− 0.004 (− 0.009; 0.001), 0.009	− 0.004 (− 0.009; − 0.000), 0.02*
Respiratory rate (frequency)	0.013 (0.001; 0.025), 0.013*	0.007 (− 0.003; 0.018), 0.007
C-reactive protein (CRP, mg/L)	0.19 (0.14; 0.25), 0.15***	0.17 (0.12; 0.22), 0.15***
Lactate (mmol/L)	0.08 (0.006; 0.16), 0.017*	0.04 (− 0.02; 0.11), 0.007
Glucose level (mmol/L)	0.10 (0.00; 0.29), 0.010	0.005 (− 0.007; 0.018), 0.02
Creatinine (µmol/L)	0.004 (0.003; 0.004), 0.33***	0.003 (0.002; 0.003), 0.28***

Associations were analyzed with linear regression models and ANOVA comparisons (RETTS-A)

^*^p < 0.05; **p < 0.01; ***p < 0.001

**Table 3:** METTS triage priority and the association with 90-day mortality and TNFR1 and TNFR2 in patients with diabetes

Triage priority	90-day mortality % (deceased/all)	TNFR (ng/mL (IQR))	
		TNFR-1	TNFR-2
Red (most acute)	27.7% (13/47)	3561 (3924)	8555 (5277)
Orange	11.8% (11/93)	2595 (2758)	7316 (6398)
Yellow	9.9% (14/141)	2746 (2362)	6876 (5638)
Green (least acute)	20.0% (2/10)	3234 (3104)	8483 (5470)

Data are expressed as absolute risk % and median (IQR). Significance was tested using one-way ANOVA for sTNFR levels, and for 90-day mortality, a trend test was performed to analyze linearity between groups

### Survival analysis

Cox regression models with HR per SD increase in TNFR1 and TNFR2 are shown in Table 4, with increasing levels of TNFR1 associated with higher mortality, but not statistically significantly when also adjusting for CRP. In secondary analyses, individuals in the highest quartile of TNFR1 had a significantly elevated mortality risk compared to individuals in quartiles 1–3 even when adjusting for CRP, age- and sex-adjusted HR 2.50, 95% CI 1.32–4.74, fully adjusted 2.21, 95% CI 1.07–4.56 (Supplementary Table 1). Un-adjusted Kaplan–Meier curves also show markedly difference between the highest TNFR1 quartile in relation to the lower quartiles (Supplementary Fig. 1).

**Table 4:** The associated risk between TNF alpha receptors and 90-day mortality

	TNF alpha receptor 1	TNF alpha receptor 2
Univariate (HR per SD, 95% CI)	1.26, 1.04; 1.52*	1.16, 0.94; 1.43
Model A (HR per SD, 95% CI)	1.25, 1.04; 1.51*	1.15, 0.93; 1.43
Model B (HR per SD, 95% CI)	1.43, 1.07; 1.91*	1.14, 0.87; 1.49
Model C (HR per SD, 95% CI)	1.29, 0.94; 1.77	1.04, 0.76; 1.42

Cox regression, Model A includes sex and age; Model B includes Model A and also BMI and creatinine; Model C includes Model B and also CRP

^*^p < 0.05

## Discussion

### Principal findings

This is the first study exploring the possible association between TNFR levels and mortality in a clinical material of patients with diabetes seeking care at a hospital ED. In this observational study, we found that patients with diabetes higher levels of TNFR1 showed a significantly higher 90-day mortality risk, however not when adjusting for CRP, while in a secondary analysis of patients with the highest quartile of TNFR1 still showed a significantly higher risk vs the three lower quartiles even after this adjustment.

### Comparison with the literature

Regarding earlier shown effects of TNFR, previous studies have found levels of TNFR1 to be of importance for both worsened kidney function and mortality in elderly patients in general [4, 5], as well as in patients with diabetes [6, 7]. The specific group studied in the present study, i.e., acute ill patients with diabetes admitted to the emergency department, is an especially frail group, as these patients have a high presence of cardiovascular diseases and acute dyspnea. Furthermore, acute dyspnea is an important and serious symptom among patients admitted to the ED [11] and could be sign of different conditions, such as heart failure, pulmonary embolism, COPD, pneumonia and other serious infections. Patients with diabetes and a high presence of cardiovascular complications are at high risk of acute infections. A rapid and correct handling and treatment of patients with dyspnea are of the highest priority, as acute dyspnea in itself is a predictor of increased mortality [12]. Furthermore, different conditions may be at hand simultaneously, especially among elderly patients with a higher risk of comorbid conditions [13]. A biomarker with the ability to predict survival in patients with dyspnea as well as in patients with diabetes regardless of type is warranted.

### Potential mechanisms

An increased mortality risk of higher levels of TNFR1 has earlier been found in individuals with and without systemic inflammation [5]. The fact that the model with adjustment for CRP lost statistical significance indicates that the inflammatory properties of TNFR1 shared with CRP carried the prognostic information. This was also supported by the secondary analysis, where the highest quartile of TNFR1 still showed significantly higher mortality risk when adjusting for CRP, although with a lower estimate. Patients with acute dyspnea due to an infection could be expected to show higher circulating TNFR1 and CRP levels.

### Clinical implications

Even if our results are of interest, it is important to confirm them in further studies, to be able to judge the clinical value. Although the clinical value based on the present results seems limited, our results could implicate TNFR1 as a potential complement to standard triage scoring among severely ill elderly patients with diabetes. In the green triage group, the smallest one with ten patients and two deaths, the higher mortality could be due to coincidence. It is notable that the TNFR values within this group were almost as high as in the red group. In an earlier study of elderly trauma patients at ED, it is found that they are managed differently to younger patients, possibly owing to under-triage by RETTS-A [14].

### Limitations and strengths

There are some limitations of the study. The study sample is small and limited to patients with diabetes and acute dyspnea seeking care at a hospital ED. However, the study is similar to other studies with patients seeking care at ED, with similar mean age [14, 15]. The number of patients was very low in the green triage group, only ten patients and with two deaths, why these findings could be by coincidence. Besides, we cannot generalize to other age or ethnic groups or to other clinical settings. Only one blood sample was drawn, and comparisons between the state before and after the admittance to the ED cannot be explored. We analyzed data on TNFR1 in quartiles which may be discussed. However, when looking at the Kaplan–Meier curve for TNFR1, this may certainly be justified. Furthermore, conclusions on causality cannot be drawn based on our observational data. It would also have been of value to have data on natriuretic peptide markers in the present study, to show if TNFR1 and TNFR2 are adding clinically important information, as natriuretic peptides are clinically used biomarkers in the ED for different conditions [16–20]. Among strengths of the study are the longitudinal study design and the characterization of study participants.

## Conclusions

We found that patients with diabetes and increased levels of TNFR1 showed a significantly higher 90-day mortality risk compared to the lower quartiles, however not when adjusting for CRP, although still significant in the highest quartile of TNFR1 vs the three lower quartiles. Even if the results are weak and in a selected patient cohort, i.e., mostly hypothesis generating, and are of no clinical relevance, it must be emphasized that it is the first of its kind to evaluate TNFR in this context with patients with diabetes seeking care at a hospital ED.

## Electronic supplementary material

Below is the link to the electronic supplementary material.

Supplementary file1 (DOCX 3379 kb)

## Abbreviations

ED: Emergency department
METTS-A: Medical Emergency Triage and Treatment System-Adult score
TNFR1: TNF alpha receptor 1
TNFR2: TNF alpha receptor 2
