# Supplementary material for: TNFR1 is associated with short-term mortality in patients with diabetes and acute dyspnea seeking care at the emergency department
Source: Acta Diabetol. 2020 Apr 12;57(10):1145–50. doi: 10.1007/s00592-020-01527-3 (PMC7496043; doi:10.1007/s00592-020-01527-3)
Supplement: Supplementary file 1 — Supplementary file1 (DOCX 3379 kb) [file 592_2020_1527_MOESM1_ESM.docx]

**Supplementary Table 1.** **The associated risk between TNF-alpha receptor 1, 4^th^ quartile against 1-3 ^th^ quartile, and 90-day mortality.** Cox regression, Model A included: Sex, Age, Model B includes Model A and also BMI, Creatinine. Model C includes Model B and also CRP **: p<0.01, *<0.05,

|  | TNF alpha receptor 1 |
| --- | --- |
| Univariate  (HR, 95 % CI) | 2.50, 1.32;4.74** |
| Model A  (HR, 95 % CI) | 2.43,1.27;4.65** |
| Model B  (HR, 95 % CI) | 2.56,1.27;5.15** |
| Model C  (HR, 95 % CI) | 2.21, 1.07;4.56* |

**Supplementary Figure 1 Kaplan Meier curve, quartiles of TNF alpha receptor 1 (a) and TNF alpha receptor 1 (b) in patients with diabetes.**

**
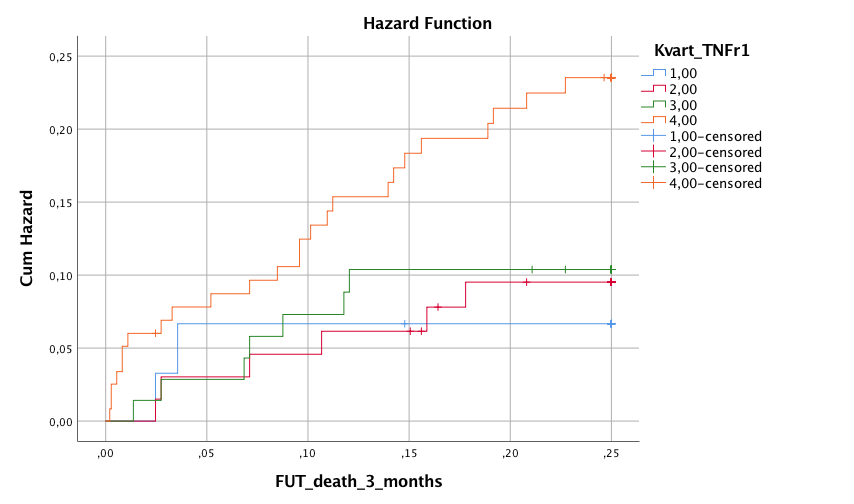
**

**
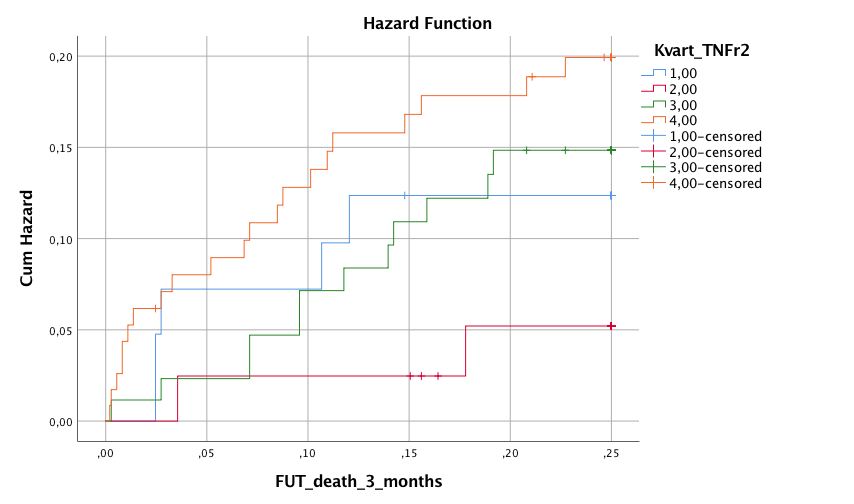
**
